# Interim FDG-PET Scan in Hodgkin's Lymphoma: Hopes and Caveats

**DOI:** 10.1155/2011/430679

**Published:** 2010-12-27

**Authors:** M. André, T. Vander Borght, A. Bosly

**Affiliations:** ^1^Department of Hematology, Grand Hôpital de Charleroi, Grand Rue, 3, 6000 Charleroi, Belgium; ^2^Department of Nuclear Medecine, Université Catholique de Louvain, Cliniques Universitaires UCL de Mont Godinne, Yvoir, Belgium; ^3^Department of Hematology, Université Catholique de Louvain, Cliniques Universitaires UCL de Mont Godinne, Yvoir, Belgium

## Abstract

FDG-PET has recently emerged as an important tool for the management of Hodgkins lymphoma. Although its use for initial staging and response evaluation at the end of treatment is well established, the place of interim PET for response assessment and subsequent treatment tailoring is still quite controversial. The use of interim PET after a few cycles of chemotherapy may allow treatment reduction for good responders, leading to lesser treatment toxicities as well as early treatment adaptation for bad responders with a potential higher chance for cure. Interpretation of interim PET is a rapidly moving field. Actually, visual interpretation is preferred over quantitative interpretation in this situation. The notion of minimal residual uptake emerged for faint persisting FDG uptake, but has evolved during the recent years. Guidelines using mediastinum and liver as references have been proposed at the expert meeting in Deauville 2009. Actually, several trials are ongoing both for localised and advanced disease to evaluate the FDG-PET potential for early treatment monitoring and tailoring. Until the results of these prospective randomized trials become available, treatment changes according to the interim PET results should remain inappropriate and limited to well-conducted clinical trials.

## 1. Introduction

This paper presents the latest evidence for the use and interpretation of early interim 18F-fluorodeoxyglucose positron emission tomography (PET) in classical Hodgkin's lymphoma (cHL).

The treatment of cHL is classically based on Ann Arbor staging. 

Patients with limited disease (stage I-II) receive combined modality treatment mostly consisting of a few cycles of adriamycin, bleomycin, vinblastine and dacarbazine (ABVD), followed by involved-field radiotherapy 20–30 Gy [1]. This approach leads to very high event-free and overall survival (10-year overall survival estimates ranging from 84 to 97%). However, these young patients experience late toxicities, such as secondary tumours and cardiac events, mostly related to radiotherapy, which lead to a delayed mortality [2]. Different randomised trials attempted to withdraw radiotherapy for unselected limited-disease patients. All of them showed reduced event-free survival for non irradiated patients [3–5]. Two recent GHSG (German Hodgkin Study Group) trials demonstrated that it is, nevertheless, possible to reduce the dose from 30 to 20 Gy in unselected patients with favourable disease [6, 7]. A concomitant expected decrease in late toxicities has not been observed yet, and our final goal remains to avoid radiotherapy for selected cHL patients.

Patients with advanced disease (bulky stage II with B symptoms and stages III-IV) receive generally 6–8 cycles of chemotherapy, mainly ABVD or BEACOPPesc. Their 10-year overall survival range from 75% to 85% [8]. If there are some evidence that BEACOPPesc is more effective than ABVD [9], this regimen also shows increased immediate (haematological) and delayed (fertility, myelodysplasia, acute myeloid leukemia) toxicities. It would be therefore, of major interest to be able to distinguish patients who can be cured with only a few cycles of BEACOPPesc or ABVD from patients who need a full course of 6–8 cycles of BEACOPPesc or even more aggressive treatments with high-dose chemotherapy and stem cell transplantation upfront.

Over the last decade, FDG-PET has become an important component in staging and end of treatment response assessment of patients with cHL [10]. When PET is combined with a CT scan (PET/CT), PET readings improve, actually PET/CT is the standard [11]. The adjunct of IV contrast-enhanced CT is likely to enhance the benefit even further [12], but this is debated as other authors report that in patients undergoing PET/CT for staging or restaging after therapy of lymphoma, diagnostic CT with IV contrast does not add useful information regarding extent of lymphoma if the low-dose CT scan is interpreted individually [13]. The spread of PET/CT is rapidly expanding, and its use should be carefully evaluated. Its role to evaluate residual masses at the end of treatment is firmly established and has become a standard of care. For staging, FDG-PET leads 15%–20% and 5%–15% changes in stage and treatment, respectively [14]. The impact of these treatment modifications has, however, never been evaluated prospectively. The use of FDG-PET for radiotherapy planning in Stage I or II cHL may induce significant irradiated field modifications, but today there are no validated guidelines to integrate such information in the treatment planning [15, 16]. Finally, FDG-PET might be used during therapy as a predictor to treatment response.

## 2. Interim PET As Predictor of Relapse

In two preliminary prospective studies, Hutchings et al. [17, 18] showed that patients with advanced cHL who were PET positive after 2 ABVD had a 2-year PFS of 0%–6% by contrast to 94% for those with PET negativeresult. Those two cohorts were joined and expanded (*n* = 260) to report the same results [19]. The prognostic value of the interim PET completely overshadowed the International Prognostic Score (IPS).

In a study of 41 patients including 23 cHL, Kostakoglu et al. performed FDG-PET soon after 1 cycle of chemotherapy and showed a 2-year progression-free survival for PET-negative patients after 1 cycle of therapy of 100.0%, compared with only 12.5% (95% CI: 2.1–32.8) in those with a positive result [20]. The timing of treatment assessment may be critical, especially to distinguish patients refractory to first-line therapy from those who relapse at a later time. The former may benefit from very early alternative therapy avoiding the complications of continued ineffective therapy. Likewise, the identification of patients who are likely to be cured by the first-line therapy may offer the potential of shortening the duration or intensity of treatment.

In a recently published metaanalysis, Terasawa [21] showed that in 360 advanced cHL, FDG-PET for interim response assessment had an overall sensitivity of 0.81 (95% CI: 0.72–0.89) and a specificity of 0.97 (95% CI: 0.94–0.99). Because of a 28.4 positive likelihood ratio, positive FDG-PET results after a few cycles of chemotherapy would probably have an excellent ability to predict poor responders in advanced adult cHL. Meta-regression and subgroup analysis did not identify factors that affect prognosis accuracy. 

The positive predictive value (PPV) of FDG-PET in localised cHL is less firmly established. In the study of Hutchings et al. [22], a significant proportion of interim PET-positive patients did not relapse. In order to identify the group of patients at very high risk for relapse more accurately, they analysed separately the patients with stage I-II and stage III-IV. The differences in PFS between stage I-II positive and negative was significant, but only 2/7 interim PET-positive patients with early stage have relapsed in contrast to advanced-stage patients who all relapsed within 2 years in the same circumstances. More recently, Sher et al. reported that only 3/20 patients with positive interim FDG-PET relapse in a study population of, mostly stage I-II, cHL patients. This especially 15% PPV was, however, obtained in the context of a consolidative radiotherapy given to all patients (PET positive and negative) [23].

## 3. Interpretation of Interim PET

### 3.1. General Considerations

In cHL, the Hodgkin or Reed-Sternberg cells represents less than 1% of the lymph node population. They are surrounded by a large number of mononuclear cells that are very metabolic and responsible for FDG uptake in vivo. The metabolic viability of such environment works as an amplifier for the FDG-PET signal, but might also become negative despite the persistence of a large tumoral mass.

This anatomopathological aspect is quite different from that observed in non-Hodgkin lymphoma where neoplastic cells account for 90% of the lymph node population. Therefore, the timing for interim FDG-PET interpretation and the guidelines might differ largely between cHL and NHL, which own different biologic behaviour and response profiles.

If the predictive value of FDG-PET depends on the type of lymphoma, the type of chemotherapy used may also be of major significance. Most of the patients included in the studies were treated using ABVD. Until recently, the prognostic value of interim FDG-PET in the context of BEACOPP regimens in advanced cHL patients was not established. Avigdor et al. [24] reported a relatively high negative predictive value (NPV; 87%), but a much lower PPV (45%), for interim FDG-PET carried out after two cycles of BEACOPPesc before decreasing therapy to ABVD. These findings were indeed different from those observed during ABVD treatment. Nevertheless, the results of early FDG-PET still maintained a significant long-term prognostic role in terms of PFS presumably due to the relatively high NPV. Similarly, Gallamini et al. [25] showed that PET carried out after two cycles of BEACOPPesc retained its long-term prognostic role despite a PPV of 60%, which was also much lower than expected.

### 3.2. Visual Interpretation and Minimal Residual Uptake (MRU)

In 10% of the patients undergoing early FDG-PET, a persisting faint residual FDG uptake is recorded, most often in a site with previous bulky disease. Hutchings et al. described this new grey-zone as minimal residual disease (MRU) and defined it as a FDG uptake just above background, which is unlikely to represent persisting cHL [22]. MRU probably represents an inflammatory reaction consecutive to chemotherapy with a nonspecific uptake of FDG. However, the boundaries of the MRU concept have evolved during the recent years. Gallamini et al. [19] considered MRU as a weakly persisting uptake with an intensity equal or slightly more than the mediastinal blood pool. In 2008, Barrington et al. proposed a residual uptake with an intensity lower or equal to the intensity of the liver [26]. The goal of the evolution of MRU definition is to reduce the false positive report. The consequence of such evolution is, however, that different MRU definitions are used among several trials making comparison extremely hazardous.

### 3.3. Quantitative Interpretation

Besides visual inspection of PET images, semiquantitative analyses using standardized uptake values (SUVs) allow for an objective assessment of treatment response, thereby eliminating observer variation and providing the opportunity to reduce the grey-zone by adding the quantitative power of PET.

However, for multicentre studies, the comparison of SUV results obtained in different centres is still hampered by the wide variability in the methodology of data acquisition, image reconstruction, and data analysis procedures. Therefore, protocols are established for standardisation and quantification of PET [27]. The quantitative assessment of tumour responses and the comparison among studies require rigorous quality control, especially if performed on different PET systems or using different protocols, and because of variation among institutions [28].

Current guidelines suggest that a visual assessment of PET status is adequate and sufficient for a positive or negative decision after completion of therapy; however, during treatment or in clinical trials, some form of semiquantitation may be helpful [29–31]. Cut-off levels of SUV to determine response to therapy are also likely to be dependent on tumour and treatment type, and so, need to be evaluated in further prospective clinical trials.

### 3.4. Proposed Guidelines for Interpretation

With the demonstration of interim FDG-PET prognostic value in cHL, different trials have been initiated to use such information for individual treatment adaptation. A standardization effort of FDG-PET interpretation criteria seems particularly relevant for a potential extension of the method on a worldwide basis.

In 2007, the International Harmonization Project (IHP) subcommittee developed consensus recommendations in the use of FDG-PET in lymphoma based on the literature and the collective expertise of its members [29]. Visual assessment alone was considered adequate for FDG-PET reading after the completion of therapy. Mediastinal blood pool activity was recommended as the reference background activity to define FDG-PET positivity for a residual mass ≥2 cm in greatest transverse diameter, regardless of its location. A smaller residual mass or a normal-sized lymph node should be considered positive if its activity is above that of the surrounding background. Specific criteria for defining FDG-PET positivity in the liver, spleen, lung, and bone marrow were also proposed. Use of attenuation-corrected PET was strongly encouraged. 

Two drawbacks can be considered for the use of IHP criteria in interim FDG-PET for cHL. First, these criteria were developed for interpretation of FDG-PET at the end of treatment and not specifically for interim FDG-PET. Moreover, the aim of PET scan performed at the end of treatment is different from that of interim scan: the first is aimed at assessing the response, the second at assessing chemosensitivity. For the former the gold standard reference is the biopsy to demonstrate CR or non-CR, for the second the gold standard does not exist, at the moment.

Second, these criteria use the lymph node size as cut-off for reference background (i.e.: when ≥2 cm: background is the mediastinal blood pool activity, whereas <2 cm it is the surrounding background). As a recent radiological study has shown the reproducibility of lymph nodes, measurements is the lowest between 15 to 20 mm, a range which might lead to major discrepancies in FDG-PET interpretation [32] as the reference background changes. In 2009, an international workshop on interim FDG-PET [33] took place in Deauville, France, to reach a consensus on simple and reproducible criteria for interim FDG-PET. The experts proposed for cHL that (1) a baseline FDG-PET/CT should be performed prior to therapy initiation, (2) and that a visual analysis using a five point-scale should be applied (Table 1), and (3) for the therapeutic decision, the cut-off should be determined according to the strategy (Tables 2(a) and 2(b)). A cohort of ABVD-treated HL patients was collected with the aim to validate the proposed criteria. This International validation study is currently under progress. 

Finally, the use of dynamic visual score, which combines the metabolic evolution of each disease site has been recently reported to be superior to the binary static scores [34].

### 3.5. FDG-PET Review System

Despite all these efforts of reading standardisation, many difficulties arise when minimal residual uptake is present. Even with blind assessment, readers may disagree especially with borderline or complex cases. Interpretation of FDG-PET is subject to a number of variables, including the reader experience. Zijlstra et al. looked at the scoring of 11 nuclear medicine physicians. They compared their results with those of an expert interpreter. The agreement was 82%–94% when the expert reading was PET-positive, but only 45% when it was PET negative. The same group also showed that more experienced readers tended to have fewer false positives, demonstrating the need for standardization [35]. 

Especially in the context of clinical trials, it is highly desirable to establish a reading procedure that synthesizes the opinions of several experts, potentially from different imaging departments, for reducing the impact of inter observer variability. A major limitation to this approach is the necessity to obtain the multiple interpretations in a clinically relevant time frame (typically less than 72 hours) in order to modify the therapeutic strategy, if needed, during the chemotherapy regimen. For these reasons, the classic retrospective or local blinded independent central review is not applicable. 

The development of such network has been recently developed by the GELA group [36]. The cornerstone of the network is a multimodality workstation which allows side-by-side display of pre- and post-treatment FDG-PET/computed tomography (CT), as well as complete image processing, including standardized uptake values analysis. A central server dispatches the raw data to the workstations of experts. They make their own independent image processing and interpretation, and send the optical scan report form with the result to the central server where an integrated computation of the interpretations is performed. From June 2007 to November 2008, FDG-PET from 166 consecutive patients included in the H10 study were reviewed. Six percent discordant PET2 readings were observed between the local site reader and the central review panel, 73% of them were modified from negative to positive. More importantly, a significantly higher inter-observer agreement (*P* ≤ .0001) was achieved when the readers could interpret side by side the PET2 with baseline FDG-PET.

Another network was also established in the United Kingdom for clinical trial in cHL. This network has been set up with a “core laboratory” to coordinate quality control and interpret scans. Images are exchanged via web on a dedicated website. Recently, a good agreement was reported within this network in a series of 44 patients with stage II–IV cHL treated with ABVD (Kappa: 0.85 (95% CI 0.74–0.96)) [37].

## 4. Ongoing Studies

Different trials evaluating FDG-PET response-adapted therapy were recently initiated (Table 3). In localised cHL, all evaluate omission of radiotherapy for FDG-PET-negative patients after 2-3 cycles of ABVD. In the EORTC-GELA-IIL trial, early modification of chemotherapy for PET-positive patients has also been a secondary objective. In advanced cHL, two different strategies have been applied: either patients who are PET-positive after 2 ABVD will receive more aggressive chemotherapy or intensification (escaladating strategy) or, in three other trials, patients who are PET-negative after 2 BEACOPPesc receive less cycles of regimen or switch to ABVD (reducing strategy).

## 5. Conclusions

Prognostic accuracy studies of interim FDG-PET for advanced-stage HL have consistently reported excellent specificity and moderately good sensitivity. 

The reported prognostic accuracy is reasonably applicable to low- or intermediate-risk advanced-stage HL patients undergoing ABVD first-line therapy. It is unclear whether the accuracy can also be applied to high-risk patients in whom BEACOPPesc is used. Moreover, the interpretation of interim FDG-PET is still the subject of debate and controversies, so that no consensus is currently reached.

Thus, the additional prognostic value of interim FDG-PET in the current management strategies is still unclear and its use should still be reserved for research settings.

## Figures and Tables

**Table 1:** Score 3 might be either considered as FDG-PET positive when a therapy decrease is planned in localised cHL or negative when treatment intensification is planned in advanced cHL.

Five Point scale.
(1) No uptake
(2) Uptake ≤ mediastinum
(3) Uptake > mediastinum but ≤ liver
(4) Uptake moderately more than liver uptake, at any site
(5) Markedly increased uptake at any site and new site of disease.

**Table:** (a)

*(1) No uptake*	
*(2) Uptake * ≤ * mediastinum*	
(3) Uptake > mediastinum but ≤ liver	
(4) Uptake moderately more than liver uptake, at any site	
(5) Markedly increased uptake at any site and new site of	
disease.	

**Table:** (b)

(1) No uptake	
(2) Uptake ≤ mediastinum	
(3) Uptake > mediastinum but ≤ liver	
*(4) Uptake moderately more than liver uptake, at any site*	
*(5) Markedly increased uptake at any site and new site of *	
* disease.*	

**Table 3:** 

Name of study	Study Group	Classification	PET intervention	Phase
20051 (H10)	EORTC-GELA-IIL	Localized	No radiotherapy PET− after 2 ABVD	III
RAPID	UK NCRI lymphoma group	Localized	No radiotherapy PET− after 3 ABVD	III
HD16	GHSG	Localized	No radiotherapy PET− after 2 ABVD	III
PET adapted chemo	GITIL	Advanced	BEACOPPesc if PET+ after 2 ABVD	III
RATHL	UK NCRI lymphoma group	Advanced	BEACOPPesc if PET+ after 2 ABVD	II
HD0801	IIL	Advanced	Salvage if PET+ after 2 ABVD	III
HD18	GHSG	Advanced	BEACOPPesc reduced to 4 cycles if PET2−	III
AHL2011	GELA	Advanced	BEACOPPesc reduced to ABVD if PET2−	III
Avigdor et al. [24]	Israel	Advanced	Reduce to 4 ABVD if PET− after 2 BEACOPPesc	II
